# MetaQuery: a web server for rapid annotation and quantitative analysis of specific genes in the human gut microbiome

**DOI:** 10.1093/bioinformatics/btv382

**Published:** 2015-06-22

**Authors:** Stephen Nayfach, Michael A. Fischbach, Katherine S. Pollard

**Affiliations:** ^1^Integrative Program in Quantitative Biology, Gladstone Institutes, and Division of Biostatistics, University of California San Francisco and; ^2^Department of Bioengineering and Therapeutic Sciences and California Institute for Quantitative Biosciences, University of California San Francisco, San Francisco, CA, USA

## Abstract

**Summary:** Microbiome researchers frequently want to know how abundant a particular microbial gene or pathway is across different human hosts, including its association with disease and its co-occurrence with other genes or microbial taxa. With thousands of publicly available metagenomes, these questions should be easy to answer. However, computational barriers prevent most researchers from conducting such analyses. We address this problem with MetaQuery, a web application for rapid and quantitative analysis of specific genes in the human gut microbiome. The user inputs one or more query genes, and our software returns the estimated abundance of these genes across 1267 publicly available fecal metagenomes from American, European and Chinese individuals. In addition, our application performs downstream statistical analyses to identify features that are associated with gene variation, including other query genes (i.e. gene co-variation), taxa, clinical variables (e.g. inflammatory bowel disease and diabetes) and average genome size. The speed and accessibility of MetaQuery are a step toward democratizing metagenomics research, which should allow many researchers to query the abundance and variation of specific genes in the human gut microbiome.

**Availability and implementation:** http://metaquery.docpollard.org.

**Contact:** snayfach@gmail.com

**Supplementary information:** Supplementary data are available at *Bioinformatics* online.

## 1 Introduction

A number of large-scale shotgun metagenomics projects have been made publicly available, enabling researchers to investigate the functional composition of microbial communities from the human body and how microbial functions correlate with disease or other traits (Aagaard *et al.*, 2013; Li *et al.*, 2014). A common goal of many microbiome studies is to quantify the abundance of specific genes across these publicly available datasets. In most cases, this task involves (1) downloading metagenomes from public repositories, (2) mapping reads to a reference database and (3) estimating gene abundances. For example, this was the approach used by Donia *et al.* (2014) to estimate the abundance of 14 000 biosynthetic gene clusters in the human microbiome. However, this approach is time-consuming and computationally demanding—requiring large amounts of storage space and processing power—and is therefore not practical for many research groups.

In an attempt to address this issue, several microbiome studies have made their functional annotations publicly available. For example, the Human Microbiome Project (HMP) Data Analysis and Coordination Center provides the abundance of KEGG Orthology Groups across 649 metagenomes from the human microbiome. Other studies have provided similar resources for other samples and databases. While useful, these resources only represent a small proportion of available samples and their annotations typically cover only a small fraction of genes in most metagenomes. For example, only 36% of reads from the HMP were mapped to a KEGG Orthology Group (Abubucker *et al.*, 2012). Furthermore, databases such as KEGG use unsupervised methods to cluster genes into ortholog groups that may not track with protein function (Schnoes *et al.*, 2009).

More recently, there have been several efforts to create comprehensive gene catalogs that cover a much higher proportion of genes in the gut microbiome. Most notably, Li *et al.* (2014) used 1267 samples from six different studies together with 511 genomes from gut microbiota to assemble a gene catalog of 9.9 million non-redundant genes. MetaQuery leverages this existing resource in order to provide users the ability to rapidly estimate the abundance of one or more query sequences across 1267 fecal metagenomes. Instead of re-mapping metagenomic reads for each query, reads were mapped once to the gene catalog, which can then be queried many times. Our framework allows the specification of sequence homology thresholds, which enable the user to define the relationship between sequence similarity and function. Finally, we use a set of 30 universal single-copy genes to normalize gene abundances to eliminate biases due to average genome size and database coverage (Manor and Borenstein, 2015; Nayfach and Pollard, 2015). This simple yet efficient framework has the potential to make large-scale metagenomics research accessible to a greater number of microbiome researchers.

## 2 Implementation

### 2.1 Gene abundance estimation

MetaQuery leverages the gene catalog and gene abundances published by Li *et al.* (2014) to rapidly estimate the abundance of one or more query genes in the human gut microbiome (Supplementary Fig. S1). First, the user submits one or more protein sequences in FASTA format, which are aligned against the gene catalog using either BLAST (Altschul *et al.*, 1990) or RAPsearch2 (Zhao *et al.*, 2012). Next, homologs of the query sequence(s) are identified in the gene catalog based on the resulting alignments and user-specified thresholds, which give the user flexibility to target either close or remote homologs of the query sequence in the gene catalog. For each query, the abundances of identified homologs are rapidly obtained from a precomputed matrix, and these abundances are summed per-query and per-sample. Next, gene abundances are optionally normalized using the relative abundance of 30 universal single-copy genes. Finally, gene abundance(s) are compared against a background set of queries in order to give the user a context in which to interpret their results.

### 2.2 Statistical analysis

After having obtained gene abundances, MetaQuery performs a number of statistical analyses. In the case of multiple query sequences, MetaQuery will build a Spearman correlation matrix of query genes across microbiome samples. Gene co-variation can identify genes that are physically linked on a genome, or genes that functionally interact in a metabolic pathway or protein complex. Next, Kruskal–Wallis tests are performed to identify genes that are differentially abundant between sample groups including: host continent (i.e. North America, Europe and Asia), and host health status (e.g. inflammatory bowel disease and diabetes). Finally, MetaQuery performs Spearman correlations of gene abundance versus average genome size (Nayfach and Pollard, 2015) and MetaPhlan (Segata *et al.*, 2012) taxonomic abundances.

## 3 Case study

We used MetaQuery to explore metagenomic variation of the fructan utilization locus found in *Bacteroides thetaiotamicron*. This locus consists of a cluster of co-regulated genes that degrade non-digestible fructose-based polysaccharides from the human diet (Sonnenburg *et al.*, 2010). We found that members of the locus tended to be quite abundant and varied extensively across gut microbiome samples, with an average estimated copy number of 1 per 50 cells, which ranked in the top 2% relative to other genes in the gene catalog. The locus was most abundant in American subjects (mean = 1 copy per 38 cells) and lowest in European individuals (mean = 1 copy per 220 cells) (Supplementary Fig. S2A). We found that the locus was marginally associated with both Crohn’s disease (*P *= 0.048) and diabetes (*P *= 0.045), indicating a potential role of microbes capable of fructan utilization in human disease (Supplementary Fig. S2B–D). Interestingly, variation of the fructan locus was strongly correlated with both AGS (*ρ *= 0.62) and the relative abundance of *Bacteroides* (*ρ *= 0.68), although even in communities with large AGS or high *Bacteroides* abundance, there was still a large variation in the abundance of the locus (Supplementary Fig. S3). Finally, we observed that the abundance of genes BT1757-58 and BT1760-63 was strongly correlated across hosts (all *ρ* > 0.97), which is consistent with the fact that these genes are physically and functionally linked (Supplementary Fig. S4).

## 4 Conclusions

MetaQuery is a web application that allows rapid and quantitative analysis of genes in the human gut microbiome. Our simple framework should enable researchers to easily investigate metagenomic variation of specific genes of interest across a large cohort of samples from the gut microbiome. Our current reference database contains genes and abundances for 1267 samples. In the future, these databases could be updated as additional fecal metagenomes become publicly available. Finally, this framework is not restricted to the human gut microbiome and could be applied to other environments, including metagenomes from soil and marine environments.

## Supplementary Material

Supplementary Data
