# Supplementary material for: MetaQuery: a web server for rapid annotation and quantitative analysis of specific genes in the human gut microbiome
Source: Bioinformatics. 2015 Jun 22;31(20):3368–70. doi: 10.1093/bioinformatics/btv382 (PMC4595903; doi:10.1093/bioinformatics/btv382)
Supplement: Supplementary Data [file supp_31_20_3368__index.html]

MetaQuery: a web server for rapid annotation and quantitative analysis of specific genes in the human gut microbiome — MetaQuery: a web server for rapid annotation and quantitative analysis of specific genes in the human gut microbiome — Supplementary Data 

# MetaQuery: a web server for rapid annotation and quantitative analysis of specific genes in the human gut microbiome

## Supplementary Data

files

- Supplementary Data - docx file
