# Supplementary material for: MetaQuery: a web server for rapid annotation and quantitative analysis of specific genes in the human gut microbiome
Source: Bioinformatics. 2015 Jun 22;31(20):3368–70. doi: 10.1093/bioinformatics/btv382 (PMC4595903; doi:10.1093/bioinformatics/btv382)
Supplement: Supplementary Data [file supp_btv382_MetaQuery_supplemental.docx]

**Supplementary Information**

**Figure S1. Workflow for estimating the abundance of a query sequence across 1,267 publicly available faecal metagenomes from human subjects. 1)** The user enters one or more protein sequences in FASTA format. These sequenced are searched against the integrated catalog of reference genes in the human gut microbiome (IGC) (Li, et al., 2014). The IGC is composed of 9.9 million genes that originate from microbial reference genomes and extensive metagenomic assemblies. Alignment is performed using either BLAST (Altschul, et al., 1990) or RAPsearch2 (Zhao, et al., 2012), as determined by the user. **2-3)** Homologs of the query sequence are identified in the IGC based on the BLAST/RAPsearch2 alignments and the set of alignment parameters entered by the user. These parameters include maximum E-value and minimum %ID in addition to a 70% minimum alignment coverage threshold. Because over 40% of the genes in the IGC lack either start/stop codons (Li, et al., 2014), many alignments will fail to globally cover both the query and target sequence. Therefore we enforce a *glocal* alignment coverage defined as: max(L_aln_/L_query_, L_aln_/L_target_), where L_aln_ is the alignment length, L_query_ is the length of the query, and L_target_ is the length of the target. **4)** Next, we obtain the relative abundances of identified homologs from a precomputed abundance matrix built by (Li, et al., 2014). This matrix consists of relative abundances of 9.9 million genes across 1,267 samples, where the relative abundance of genes is scaled to sum to 1.0 per-sample. For each query, we sum the relative abundances of identified homologs for each sample. **5)** Optionally, our software normalizes gene relative abundances using a panel of 30 universal single-copy genes (Nayfach and Pollard, 2015). The result of this normalization is a metric called Average Genomic Copy Number, which represents the estimated average copy number of a gene across microbial cells (Manor and Borenstein, 2015). Without normalization, the resulting metric is Relative Abundance, which is scaled to sum to 1.0 across all genes for a sample.

| **Percent Identity Between Query and Target** | | **Fraction of BLAST Alignments Reported** |
| --- | --- | --- |
| Lower bound | Upper bound | RAPsearch2 |
| 20 | 30 | 0.151 |
| 25 | 35 | 0.299 |
| 30 | 40 | 0.543 |
| 35 | 45 | 0.735 |
| 40 | 50 | 0.848 |
| 45 | 55 | 0.909 |
| 50 | 60 | 0.962 |
| 55 | 65 | 0.979 |
| 60 | 70 | 0.983 |
| 65 | 75 | 0.990 |
| 70 | 80 | 0.994 |
| 75 | 85 | 0.995 |
| 80 | 90 | 0.995 |
| 85 | 95 | 0.989 |
| 90 | 100 | 0.988 |

**Table S1. Sensitivity of RAPsearch2 relative to BLASTP.** 100 protein sequences were randomly selected from the reference genome *Bacteroides vulgatus* ATCC 8482 and searched against the IGC using BLASTP (v2.2.25) and RAPsearch2 (v2.22). All alignments with an E-value < 1e-5 were reported. RAPsearch2 was run with default parameters, except –v to report up to 10,000 alignments per query. Likewise, BLASTP was run with defaults, except max_target_seqs to report up to 10,000 alignments per query.

|  | **Fold Increase in Speed vs. BLAST using 10 CPUs** | | |
| --- | --- | --- | --- |
| **Search Tool** | **1 query** | **10 queries** | **100 queries** |
| RAPsearch2 | 0.29 | 2.32 | 14.68 |

**Table S2. Speed of RAPsearch2 relative to BLASTP.** BLASTP and RAPsearch were run using the same query and reference sequences described in Table S1. Each program was run using between 1-100 queries. The table shows the increase in speed of RAPsearch2 relative to BLASTP.

|  | Plotting OFF | | | |
| --- | --- | --- | --- | --- |
| Module | 1 query | 10 queries | 100 queries | 1,000 queries |
| Abundance estimation (seconds) | 18.4 | 32.9 | 80.2 | 406.8 |
| Abundance summary (seconds) | 0.6 | 0.6 | 1.0 | 11.9 |
| Phenotype associations (seconds) | 0.5 | 0.6 | 2.0 | 19.9 |
| Covariation with AGS (seconds) | 0.5 | 0.6 | 1.0 | 7.8 |
| Gene-Gene covariation (seconds) | n/a | 0.3 | 0.4 | 6.6 |
| Taxonomic covariation (seconds) | 4.7 | 5.4 | 27.1 | 255.9 |
| Total time (seconds) | 24.7 | 40.4 | 111.7 | 708.9 |
| Total disk space (Mb) | 0.0 | 0.2 | 2.3 | 28.0 |
|  |  |  |  |  |
|  | Plotting ON | | | |
| Module | 1 query | 10 queries | 100 queries | 1,000 queries |
| Abundance estimation (seconds) | 19.9 | 30.2 | 52.7 | 206.2 |
| Abundance summary (seconds) | 0.6 | 1.2 | 3.9 | 7.7 |
| Phenotype associations (seconds) | 0.6 | 1.2 | 7.0 | 67.1 |
| Covariation with AGS (seconds) | 0.6 | 1.0 | 4.5 | 41.3 |
| Gene-Gene covariation (seconds) | n/a | 0.3 | 0.7 | 17.4 |
| Taxonomic covariation (seconds) | 4.7 | 6.0 | 27.4 | 240.3 |
| Total time (seconds) | 26.4 | 39.8 | 96.2 | 580.1 |
| Total disk space (Mb) | 0.3 | 2.4 | 21 | 178 |

**Table S3. Runtime and disk-space benchmark.** MetaQuery was run using different numbers of query sequences (1 to 1,000) and with plotting turned on/off. For all tests, MetaQuery was run in multi-query mode, using RAPsearch2 (faster/less sensitive), with default alignment parameters, and Average Genomic Copy Number was the metric used to estimate gene abundances. The tables above show the runtime of MetaQuery as a function of query size and plotting on/off. Total disk space is the number of megabytes needed to store compressed, downloadable results.

C

B

D

A

**Figure S2. Differential abundance of the Fructan-utilization locus.** MetaQuery was used to estimate the abundance of the *Bacteroides thetaiotamicron* Fructan polysaccharide utilization locus (Sonnenburg, et al., 2010) across 1,267 faecal metagenomes. We uploaded the 9 genes within the locus (BT1754, BT1757-BT1763, BT1765) to MetaQuery and ran the application using the mode “Per Query Abundance”, which estimates the abundance of each query independently. We applied a 90% identity threshold, in order to identify close homologs of the locus genes and used the abundance metric “Average Genomic Copy Number”, which is obtained by normalizing gene abundances using a panel of universal single-copy genes. Kruskal-Wallis tests were performed to determine whether the estimated median abundance of the locus differed significantly between groups. For each analysis, P-values were corrected for multiple testing using the Benjamini-Hochberg procedure. The percentile indicates the rank of each P-value relative to a set of randomly selected genes from the IGC run with MetaQuery using matched parameters (alignment tool, e-value, %ID, normalization). All figures were generated using the mean per query abundance across the Fructan locus and can be viewed online at: <http://metaquery.docpollard.org/cgi-bin/fetch_results.py?job_id=ORhY8o>. **A)** Abundance of the Fructan locus across samples from different continents. A percentile of 2.3 indicates that the P-value of 4.7e-32 ranks in the top 2.3% relative to other genes in the IGC. **B)** Abundance of the Fructan locus across Chinese diabetics and healthy individuals. **C)** Abundance of the Fructan locus across Spanish individuals with and without Crohn’s disease. **D)** Abundance of the Fructan locus across Spanish individuals with and without Ulcerative Colitis.

**Figure S3. Covariation of the Fructan-utilization locus with AGS.** MetaQuery was used to estimate the abundance of the Fructan polysaccharide utilization locus as described in the caption of Figure S2. Spearman correlations were computed for Average Genome Size (AGS) versus the estimated abundance of the Fructan-utilization locus. The percentile indicates that a P-value of 1.8e-131 ranks in the top 0.2% relative to a set of randomly selected genes from the IGC run with MetaQuery using matched parameters (alignment tool, e-value, %ID, normalization). The figure was automatically generated by MetaQuery and can be viewed online at: <http://metaquery.docpollard.org/cgi-bin/fetch_results.py?job_id=ORhY8o>.


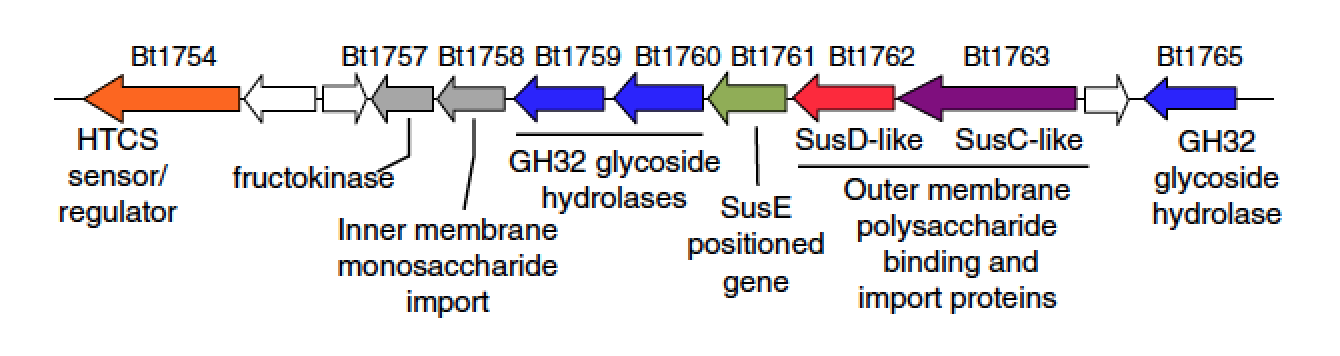


A

B

**Figure S4. Co-variation of genes within the Fructan-utilization locus across human faecal metagenomes. A)** Annotated genes within the *Bacteroides thetaiotamicron* fructan-utilization locus. Adapted from (Sonnenburg, et al., 2010). **B)** MetaQuery was used to estimate the abundance of 9 genes within the Fructan polysaccharide utilization locus as described in the caption of Figure S2. Spearman correlations were performed for each pair of genes across 1,267 faecal metagenomes. Numbers within each cell indicate correlation coefficients. Locus ids are indicated on rows/columns and correspond to ids from the top figure. The heatmap was automatically generated by MetaQuery and can be viewed online at: [http://metaquery.docpollard.org/cgi-bin/fetch_results.py?job_id=ORhY8o](http://metaquery.docpollard.org/cgi-bin/fetch_results.py?job_id=ORhY8o%20)

**References**

Altschul, S.F.*, et al.* Basic local alignment search tool. *Journal of Molecular Biology* 1990;215(3):403-410.

Li, J.*, et al.* An integrated catalog of reference genes in the human gut microbiome. *Nature biotechnology* 2014;32(8):834-841.

Manor, O. and Borenstein, E. MUSiCC: a marker genes based framework for metagenomic normalization and accurate profiling of gene abundances in the microbiome. *Genome biology* 2015;16(1).

Nayfach, S. and Pollard, K.S. Average genome size estimation improves comparative metagenomics and sheds light on the functional ecology of the human microbiome. *Genome biology* 2015;16(1):51.

Sonnenburg, E.D.*, et al.* Specificity of polysaccharide use in intestinal bacteroides species determines diet-induced microbiota alterations. *Cell* 2010;141(7):1241-1252.

Zhao, Y., Tang, H. and Ye, Y. RAPSearch2: a fast and memory-efficient protein similarity search tool for next-generation sequencing data. *Bioinformatics* 2012;28(1):125-126.
